# Supplementary material for: Species-Specific Impact of Fusarium Infection on the Root and Shoot Characteristics of Asparagus
Source: Pathogens. 2020 Jun 24;9(6):509. doi: 10.3390/pathogens9060509 (PMC7350344; doi:10.3390/pathogens9060509)
Supplement: Supplementary file 1 [file pathogens-09-00509-s001.pdf]

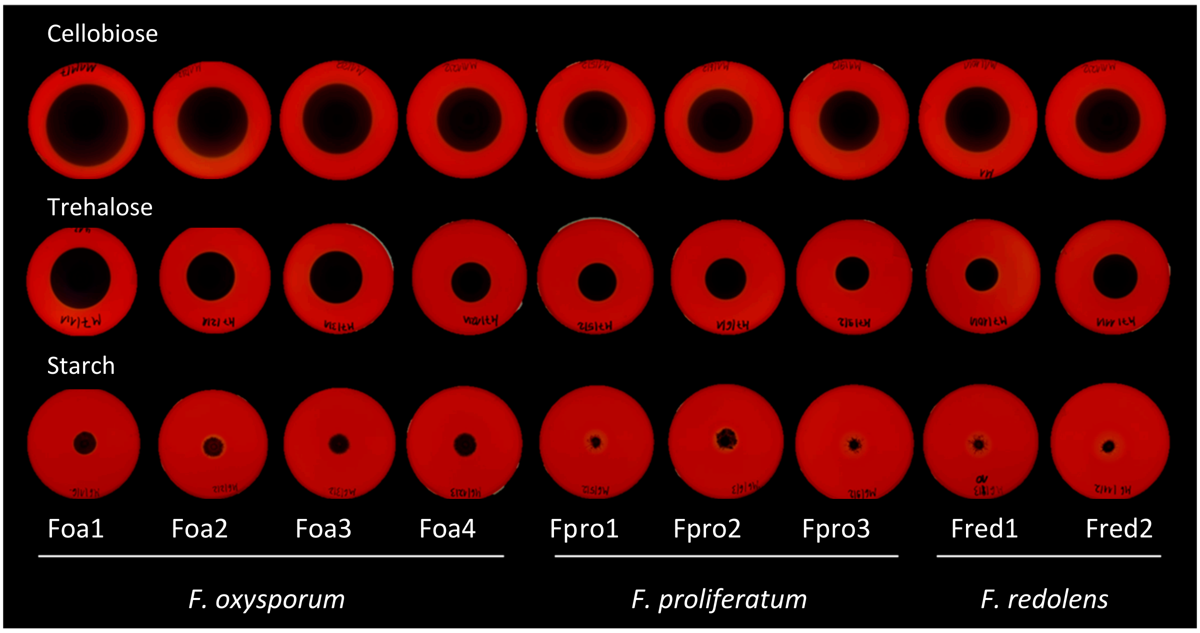

Supplementary Figure S1: Extracellular enzymatic activity of *Fusarium* spp. isolates on plates containing cellobiose, trehalose or starch.

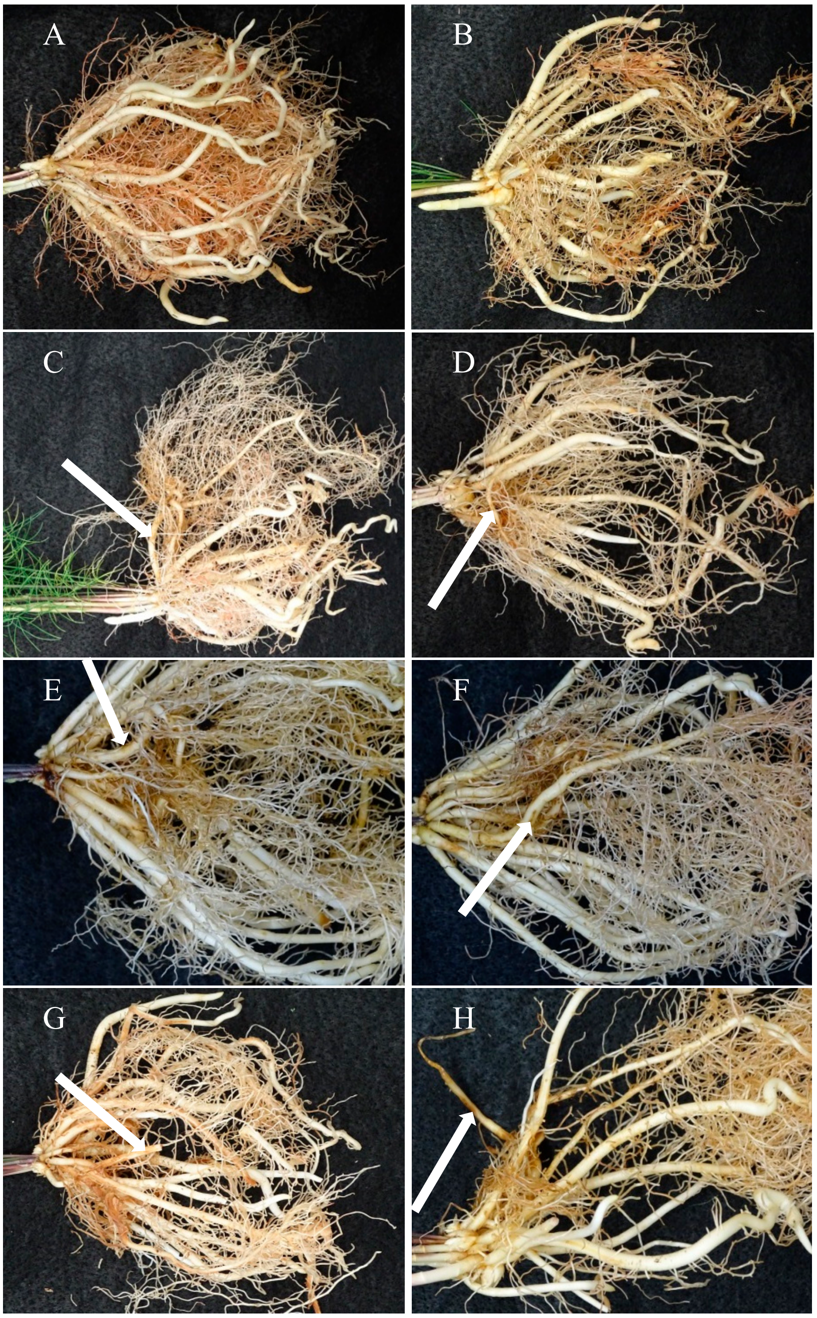

Supplementary Figure S2: Typical symptoms caused by *Fusarium* spp. isolates on roots of asparagus ‘Backlim’ and ‘Rapsody’. A, ‘Rapsody’ control; B, ‘Backlim’ control; C, ‘Rapsody’ *F. oxysporum* Foa4; D, ‘Backlim’ *F. oxysporum* Foa4; E, ‘Rapsody’ *F. proliferatum* Fpro2; F, ‘Backlim’ *F. proliferatum* Fpro2; G, ‘Rapsody’ *F. redolens* Fred1; ‘Backlim’ *F. redolens* Fred1; white arrows point at typical lesions.

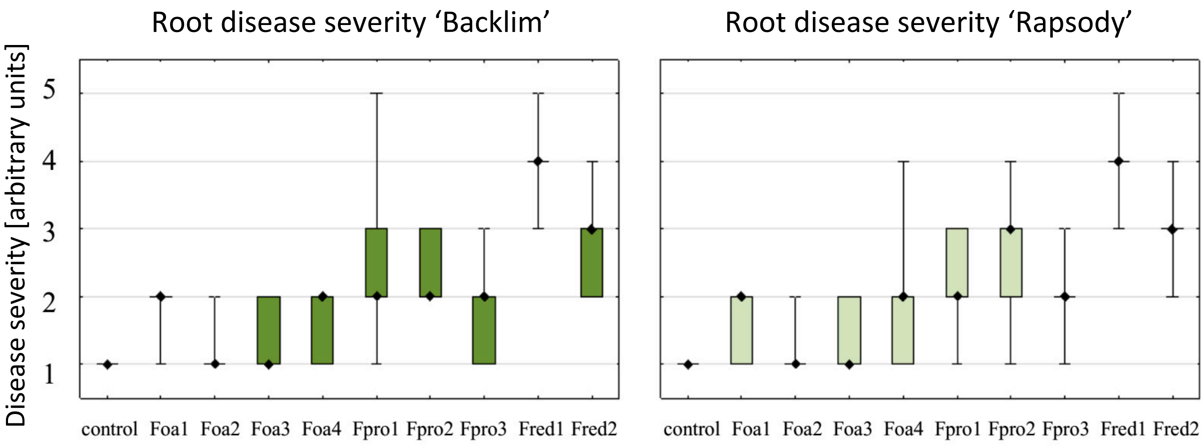

Supplementary Figure S3: Disease severity (DS) of roots of asparagus 'Backlim' and 'Rapsody'. Plants were inoculated at BBCH 12-13 with isolates of either *Fusarium oxysporum* f.sp. *asparagi* (Foa1, Foa2, Foa3, Foa4), *F. proliferatum* (Fpro1, Fpro2, Fpro3), *F. redolens* (Fred1, Fred2) with 6-8 x 10^6 conidia mL^{-1}, or with water (control), and sampled 8 weeks after inoculation (n = 9). Box plots indicate the interquartile ranges (between 25th and 75th percentile) of DS evaluation classes with marks depicting medians (♦); whiskers indicate min- and max-values assessed in each treatment.

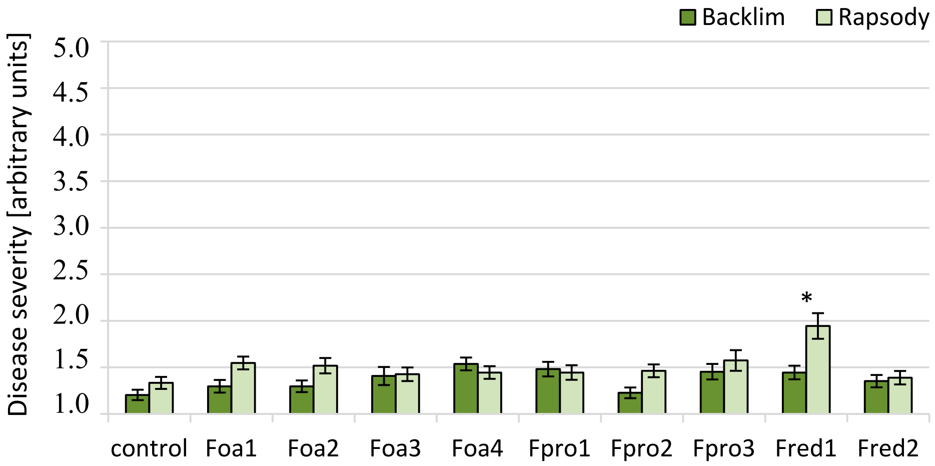

Supplementary Figure S4: Disease severity of ferns of asparagus 'Backlim' and 'Rapsody'. Plants were inoculated at BBCH 12-13 with isolates of either *Fusarium oxysporum* f.sp. *asparagi* (Foa1, Foa2, Foa3, Foa4), *F. proliferatum* (Fpro1, Fpro2, Fpro3), *F. redolens* (Fred1, Fred2) with 6-8 x 10^6 conidia mL^{-1}, or with water (control) and sampled 8 weeks after inoculation (n = 9). Values are the means ± standard errors. Effect of isolates on plants was statistically analysed over both cultivars likewise; ($P < 0.0001$; $\chi 2$ test).

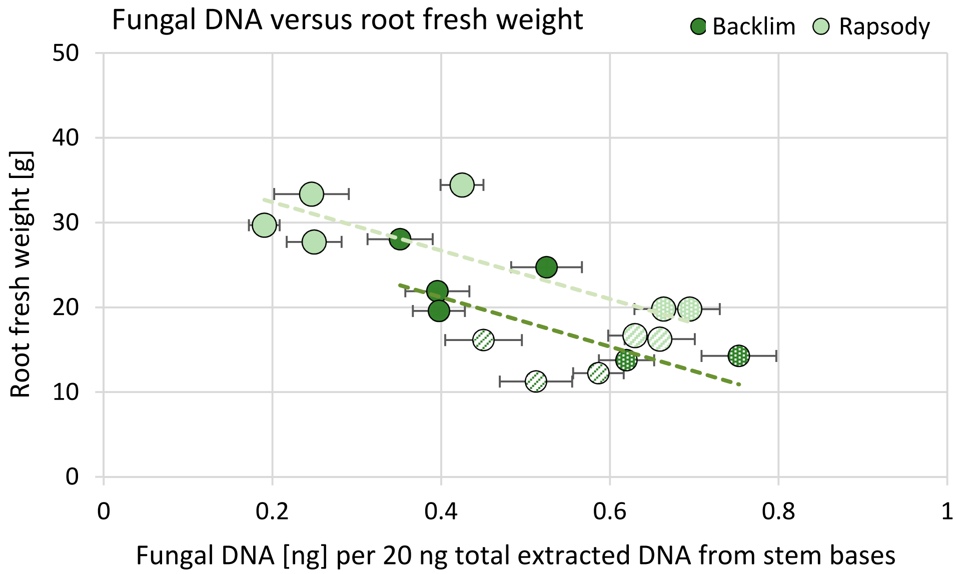

Supplementary Figure S5: Relationship between concentration of fungal DNA in stem bases of asparagus and root fresh weight after inoculation with *Fusarium* spp. Asparagus 'Backlim' (dark green) and 'Rapsody' (light green) (n = 9) after inoculation with *F. oxysporum* f.sp. *asparagi* (Foa1, Foa2, Foa3, Foa4, solid fill); *F. proliferatum* (Fpro 1, Fpro2, Fpro3, pattern fill ascending stripes) and *F. redolens* (Fred1, Fred2, pattern fill points). Values are the means ± standard errors. Data were collected from an additional experiment. Fungal DNA is given as ng in 20 ng total DNA extracted from respective tissue, root fresh weight [g].

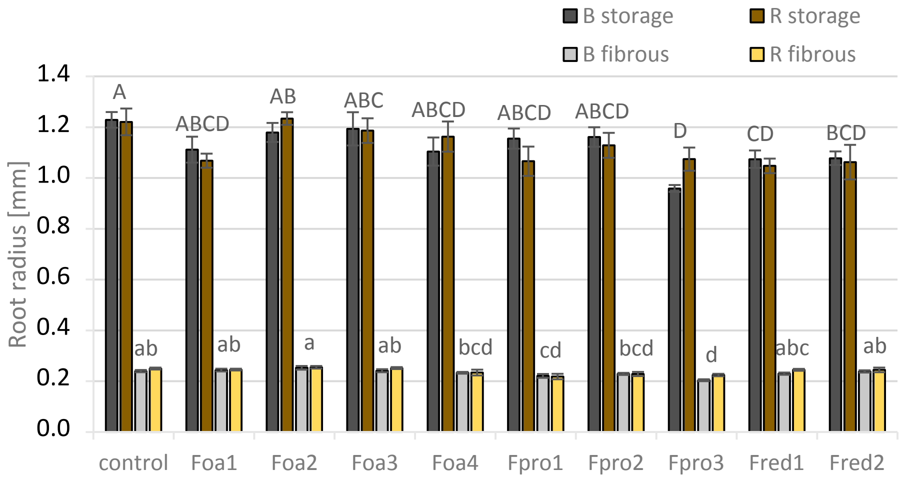

Supplementary Figure S6: Radius of storage and fibrous roots [mm] of asparagus 'Backlim' (B) and 'Rapsody' (R). Plants were inoculated at BBCH 12-13 with isolates of either *Fusarium oxysporum* f.sp. *asparagi* (Foa1, Foa2, Foa3, Foa4), *F. proliferatum* (Fpro1, Fpro2, Fpro3), *F. redolens* (Fred1, Fred2) with 6-8 x 10^6 conidia mL^{-1}, or with water (control) and sampled 8 weeks after inoculation (n = 6). Values are the means of 'Backlim' and 'Rapsody' ± standard errors. Effect of isolates on root radius of storage and fibrous roots was statistically analysed over both cultivars likewise; different letters show significance among the *Fusarium spp.* isolates and control (Tukey test; $P < 0.05$).
